# Structural and functional characterization of hMEX‐3C Ring finger domain as an E3 ubiquitin ligase

**DOI:** 10.1002/pro.3473

**Published:** 2018-10-18

**Authors:** Sayed Ala Moududee, Yiyang Jiang, Nshogoza Gilbert, Guodong Xie, Zheng Xu, Jihui Wu, Qingguo Gong, Yajun Tang, Yunyu Shi

**Affiliations:** ^1^ Hefei National Laboratory for Physical Science at Microscale and School of Life Sciences University of Science and Technology of China Hefei Anhui 230026 People's Republic of China; ^2^ CAS Center for Excellence in Bio macromolecules Chinese Academy of Sciences Beijing 100101 People's Republic of China

**Keywords:** hMEX‐3C, Ring finger domain, E3 ubiquitin ligase, ubiquitination

## Abstract

MEX‐3C, a novel RNA binding E3 ubiquitin ligases, contains two N‐terminal heterogeneous nuclear ribonucleoprotein K homology (KH) domains and C‐terminal Ring finger domain. Recent evidence has suggested that human MEX‐3C has a strong bondage with carcinogenesis and the MEX‐3C‐mediated ubiquitination of RIG‐I is essential for the antiviral innate immune response. Moreover, the Ring finger domain of MEX‐3C could regulate the degradation of *HLA‐A2* (an MHC‐I allotype) mRNA with a novel mechanism. However, the structural basis for the ubiquitination catalyzed by hMEX‐3C Ring finger domain remains evasive. In this study, we solved the crystal structure of dimeric Ring finger domain of hMEX‐3C and compared it with the complex structure of MDM2/MDMX–UbcH5b–Ub. Our ubiquitination assay demonstrated that the Ring finger domain of hMEX‐3C acts as a ubiquitin E3 ligase *in vitro*, cooperating with specific E2 to mediate ubiquitination. Then, we identified several key residues in Ring finger domain of hMEX‐3C possibly involved in the interaction with E2–Ub conjugate and analyzed the E3 ligase activities of wild type and mutants at key sites. Additionally, zinc chelation experiments indicated that the intact structural stability is essential for the self‐ubiquitination activity of the Ring finger domain of hMEX‐3C. Taken together, our studies provided new insight into the mechanism of the Ring finger domain of hMEX‐3C that may play an important role in eliciting antiviral immune responses and therapeutic interventions.

## Introduction

Ubiquitination is the process of attaching ubiquitin (Ub), a small functional regulatory protein, to another targeted molecule. It plays very important roles in many biological processes including protein cytosis, proteasomal degradation, cell‐cycle control, protein trafficking, DNA damage repair, and gene transcription.^1^, ^2^ Ubiquitination implicates the sequential action of ubiquitin‐activating enzymes (E1), ubiquitin‐conjugating enzymes (E2), and ubiquitin‐ligases (E3).^3^, ^4^, ^5^ E3 ubiquitin ligase controls efficiency, specificity, and patterns of ubiquitination. Therefore, E3 ubiquitin ligase involves in the regulation of several diseases like cancer, diabetes, neurological dysfunction, defective immunity, and dementia.^6^, ^7^

Human MEX‐3 protein, belonging to a novel subfamily of evolutionarily conserved RNA binding proteins, is the human homologs of *Caenorhabditis elegans* MEX‐3 and involved in the post‐transcriptional regulation.^8^ There are four human MEX‐3 proteins including MEX‐3A, MEX‐3B, MEX‐3C, and MEX‐3D. Each contains two heterogeneous nuclear ribonucleoprotein K homology (KH) domains at the N‐terminus and an ubiquitin E3 ligase Ring finger domain at the C‐terminus.^9^ Among these homologs, human MEX‐3C (hMEX‐3C) is broadly expressed in numerous tissues, particularly in thymus and spleen.^10^ hMEX‐3C has been reported to be associated with several diseases. For example, it is susceptible to important genetic hypertension^11^ and identified as a new chromosomal instability (CIN) suppressor in cancer.^12^ It plays an essential role in the regulation of energy balance because its mutation moderates adiposity and develops energy cost.^13^ hMEX‐3C is a unique RNA binding ubiquitin E3 ligase and plays a key role in mRNA degradation, such as post‐transcriptional regulation of common Human leukocyte antigen serotype A2 (*HLA‐A2*) allotypes. It binds the 3′UTR of *HLA‐A2* mRNA, which induces Ring finger domain dependent degradation.^14^, ^15^ It has been found that hMEX‐3C is critical for innate immune responses against RNA virus infections. Ubiquitination of Lys‐63 linked ubiquitin chains to RIG‐I by hMEX‐3C in stress granules is important for the activation of the IFNB promoter and RIG‐I‐mediated antiviral innate immunity.^10^

Ring finger domain is responsible for the ubiquitination reaction and defines the largest family of E3 ligases. Among more than 600 human E3 ligases, there are at least 15 which contain an RNA‐binding domain,^16^ in addition to the Ring finger domain required for the ubiquitination reaction.^10^ Ring finger domain contains a set of cysteine and histidine residues which have specific spacing due to their roles as two zinc ligands to stabilize a distinctive globular conformation.^17^ Recent studies have reported that the Ring finger domain binds E2–Ub thioester intermediate and promotes E2–Ub closed active conformations required for direct Ub transfer from E2 to substrate.^18^ The interaction between E2–Ub and the Ring finger domain has also been investigated by structural research.^19^, ^20^, ^21^, ^22^ Previous structural studies have reported that the Ring finger domain interacts to the E2 and positions the “donor ubiquitin” in the E2–Ub for ubiquitin transfer to a remotely bound protein substrate.^20^, ^23^, ^24^ For Ring E3 ligases, dimerization is a very important mechanism for catalysis^25^ because one Ring monomer recruits both the E2 and Ub by a conserved set of interactions and the other Ring domain interacts the same Ub using various non‐conserved residues and stabilizes ubiquitin in the closed conformation.^26^

In this study, we solved the crystal structure of the dimeric Ring finger domain of hMEX‐3C which employs a typical Ring fold and is stabilized by the coordination of two zinc ions. In addition, by superimposing the structure of the hMEX‐3C Ring homodimer with that of MDM2–MDMX–UbcH5b–Ub (PDB ID 5MNJ), a model of hMEX‐3C Ring–E2–Ub complex was proposed and several interface residues of hMEX‐3C Ring finger domain required for recruitment of E2 were, therefore, identified. To characterize the E3 ubiquitin ligase activity of hMEX‐3C Ring finger domain *in vitro*, we performed ubiquitination assays and found that it functions as an E3 ubiquitin ligase and catalyzes self‐ubiquitination. Furthermore, mutations at the possible interaction interface of hMEX‐3C with E2 mostly decreased the ubiquitination activities. Taken together, the intact hMEX‐3C Ring finger domain is responsible for the interaction with E2 enzyme and required for the ubiquitin ligase activity.

## Results

### *The overall structure of hMEX‐3C Ring finger domain*

**Figure 1:**
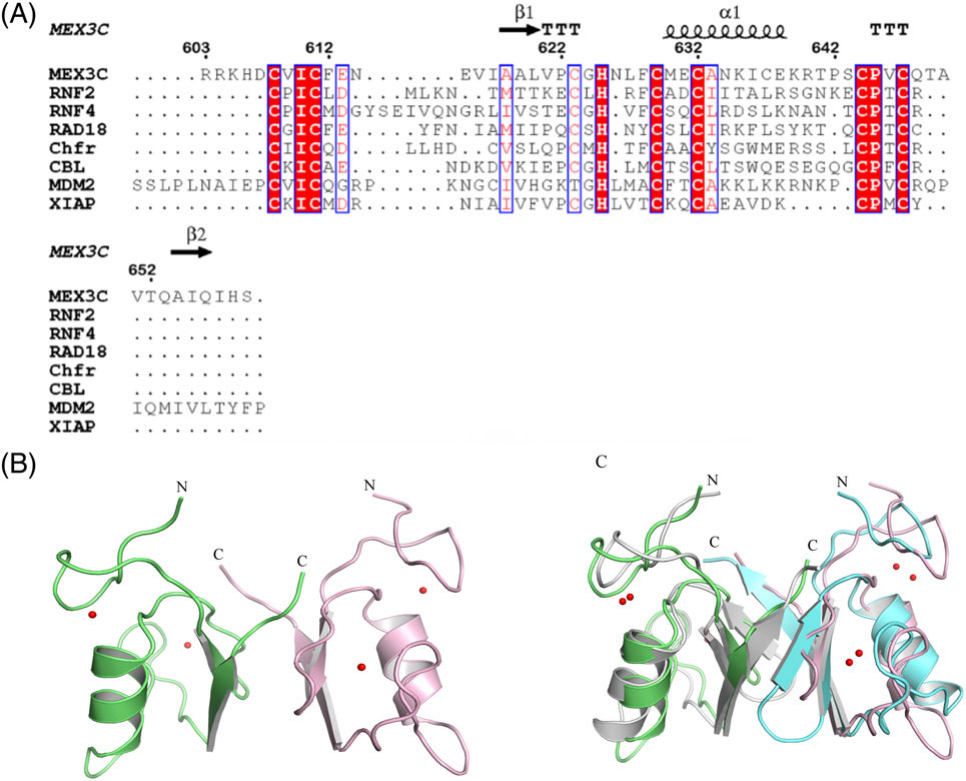
The overall structure of the Ring finger domain of hMEX‐3C. (A) Structure‐based sequence alignment of the Ring finger domain of hMEX‐3C with other Ring finger domains. Residues identical in all sequences are shown in red column with white letters and residues similar in white column red letters. Asterisks indicate the conserved residues in the Ring finger domain. (B) Ribbon presentation of the hMEX‐3C Ring finger domain dimer (Residues 605–659). Zinc ions and coordinating residues are shown as spheres and sticks, respectively. (c) Structural superimposition of the hMEX‐3C homodimer and MDM2/MDMX (PDB 2VJE) heterodimer. The hMEX‐3C homodimer are colored lime and pink and MDM2/MDMX (PDB 2VJE) heterodimer are colored gray and aquamarine, respectively.

The sequence alignment showed that the hMEX‐3C Ring finger domain shares significant homology with several known Ring finger domains [Fig. 1(a)]. The Ring finger domain can be defined by the consensus sequence CX_2_CX_(9–39)_CX_(1–3)_CX_(2–3)_C/HX_2_CX_(4–48)_CX_2_C, in which Cys and His are Zinc‐binding residues. By sequence alignment, we found that hMEX‐3C Ring finger domain has seven conserved Cys residues (C608, C611, C623, C629, C632, C644, and C647) and one His residue (H625) which are likely involved in zinc binding [Fig. 1(a)].

To understand the molecular details of the Ring finger domain of hMEX‐3C, we determined the crystal structure of Ring finger domain homodimer (Residues 605–659) at a resolution of 2.2 Å with *R*_work_ and *R*_free_ 20.7% and 24.3%, respectively. The Ring finger domain structure was refined in space group *P*1, with eight molecules per asymmetric unit. Data collection and refinement statistics are summarized in Table 1. The Ring finger domain of hMEX‐3C forms stable dimer in solution as determined using size exclusion chromatography method (Fig. S1, Supporting information). The core Ring finger domain adopts the typical Ring fold, consisting of two antiparallel β‐strands (β1 from Residues 618–621, and β2 from Residues 652–656) and one central α helix (α1 from Residues 630–638) [Fig. 1(b)]. In addition, two zinc ions (Zn^2+^) are coordinated within a C3HC4 “cross brace” manner (C608, C611, C623, H625, C629, C632, C644, and C647) by seven Cys side chains and one His side chain, which enables the correct folding and biological activity of the Ring finger domain [Fig. 1(b)]. It suggests that the two Zn^2+^ ions are very important to maintain the stability of the intact Ring finger domain structure and contribute to its E3 ligase activity.

**Table 1:** Data Collection and Refinement Statistics

Data collection statistics	MEX‐3C
PDB ID	5ZI6
Data collection	
Wavelength (Å)	0.9777
Space group	*P1*
Cell dimensions	
*a, b, c* (Å)	37.14, 44.57, 67.16
*α, β, γ* (°)	92.43 90.50 91.39
Resolution range (Å)	40.00–2.20 (2.24–2.20)
*R*_merge_ (%)	8.8 (89.8)
*I/σI*	11.97 (0.88)
*CC* ^*1/2*^	0.991/0.608
Completeness (%)	99.9 (99.9)
Redundancy	3.6 (3.1)
Wilson B‐factor (Å^2^)	42.8
Refinement	
Number of reflections (overall)	19,978
Number of reflections (test set)	960
*R*_work_/*R*_free_ (%)	20.7/24.3
Number of atoms	
Protein/ligands/water	3320/16/10
*B*‐factors (Å^2^)	
Protein/ligands/water	51.79/43.58/39.47
R.M.S. deviations	
Bond length (Å)	0.009
Bond angles (°)	1.281
Ramachandran plot (%)	
Favored/allowed/outlier (%)	95/5/0

*Values in parentheses are for the highest resolution shell.

Using Dali search for structural homologs of hMEX‐3C Ring finger domain,^27^ we found the three highest similar hits are MDM2 (*Z*‐score of 9.1, PDB code 2vje), MYLIP (*Z*‐score of 8.7, PDB code 2yhn), and XIAP (*Z*‐score of 8.3, PDB code 5o6t). The superimposition indicated that the Ring finger domain structure of hMEX‐3C closely resembles that of MDM2 (PDB 2vje) [Fig. 1(c)] suggesting that hMEX‐3C may share the same E2 enzymes with MDM2 (PDB 2vje).

### *The Ring finger domain of hMEX‐3C acts as an E3 ligase in vitro*

**Figure 2:**
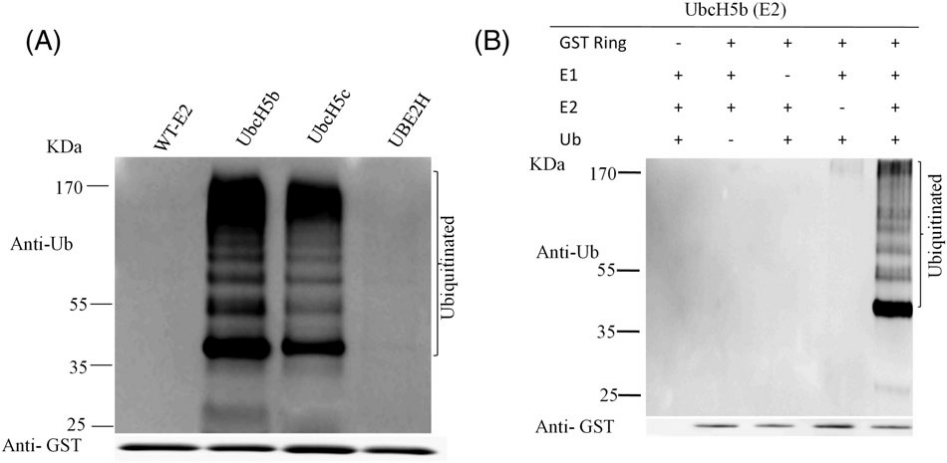
The Ring finger domain of hMEX‐3C mediates specific E2‐dependent ubiquitination. (A) Purified Ring finger domain of hMEX‐3C was incubated with E1, ubiquitin and various E2 (UbcH5b, UbcH5c, and UBE2H) (Lanes 2, 3, and 4) or without E2 enzyme (Lane 1). All reaction mixtures were kept at 37°C for 90 min. Then, reaction mixtures were fractionated by 10% SDS‐PAGE and analyzed by immunoblotting using anti‐Ub and anti‐GST antibodies. (B) Each component is required for ubiquitination. In every lane, one component was removed in each reaction. Then the reaction mixtures were resolved by 10% SDS‐PAGE and analyzed by western blot with antibodies.

The sequence conservation and structural homology of hMEX‐3C Ring finger domain with other different E3 ubiquitin ligases indicate its significant membership to E3 ubiquitin ligase category [Fig. 1(a)]. Then, we performed *in vitro* ubiquitination assay to determine the activity of hMEX‐3C Ring finger domain. In the assay, GST‐fused hMEX‐3C Ring finger domain, E1 and various E2s (UbcH5b, UbcH5c, and UBE2H) were incubated together with ubiquitin. After incubation, the reactants were determined by western blot using anti‐GST and anti‐Ub antibodies. In the presence of both E2 of UbcH5b and UbcH5c, the Ring finger domain of hMEX‐3C was multi‐ubiquitinylated [Fig. 2(a), Lane (2,3)]. While in the absence of any E2, there is no band [Fig. 2(a), Lane (1)]. Also, no multi‐ubiquitinylated band was observed with the third E2 (UBE2H) [Fig. 2(a), Lane (4)]. Taken together, all the observations showed that the Ring finger domain of hMEX‐3C displays specific E2 dependent E3 ligase activity.

To further determine the minimum requirement of the component in ubiquitination assays, we fixed UbcH5b as the E2 enzyme. Then each component in the assay including Ring finger domain of hMEX‐3C, E1, ubiquitin, and E2 (UbcH5b) was removed from the mixture, respectively. We found that, for all incomplete mixtures, there was no observed band compared with the complete reaction mixture [Fig. 2(b)]. This result revealed that each component in the ubiquitination assay is essential to the formation of multi‐ubiquitinylated products.

### *Structural superimposition and ubiquitination assays identified key residues involved in interaction with E2–Ub*

**Figure 3:**
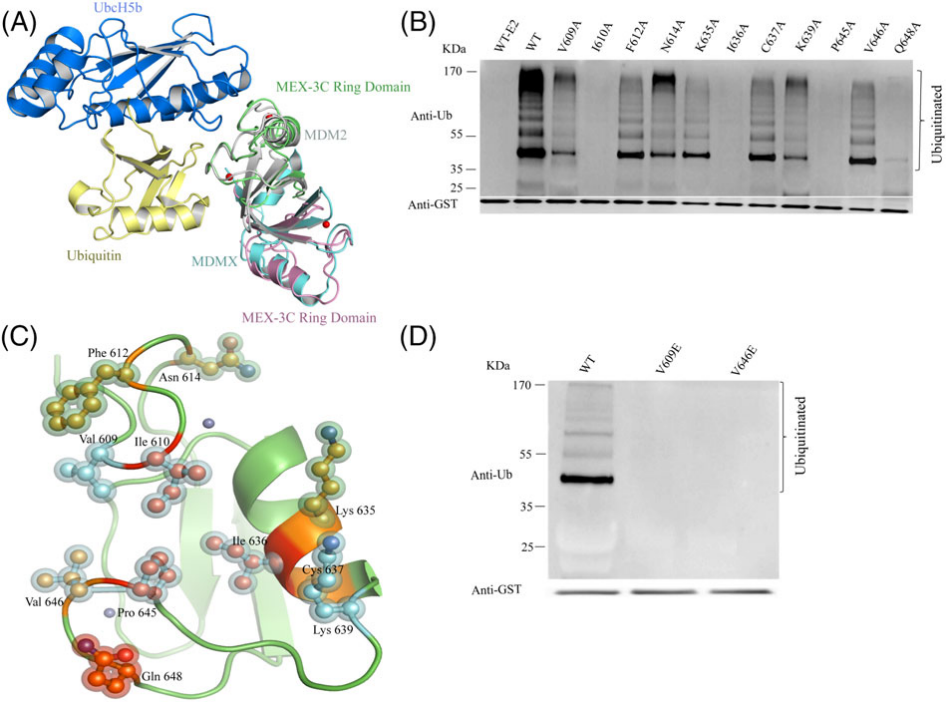
Mutations of the key residues dramatically affected the ubiquitination activity. (A) Structural superimposition of hMEX‐3C Ring homodimer (lime and pink) and complex of MDM2/MDMX‐UbcH5b‐Ub (PDB ID 5MNJ is colored gray, aquamarine, marine, and pale yellow). (B) The predicted UbcH5b–hMEX‐3C Ring finger domain interaction residues were mutated to alanine, respectively. The activities of wild‐type and mutants were investigated using “*in vitro*” ubiquitination assays. (C) A putative E2‐binding interface of the hMEX‐3C Ring finger domain. Residues within 4 Å of UbcH5b are shown in ball and stick format. According to their effect in ubiquitination activity, as determined, the side chains of mutant residues are colored differently (wild‐type activity in yellow, partial activity in cyan, and inactive activity in red). (D) Mutants of V609E and V646E are shown inactive in ubiquitination assay.

As shown in Figure 2(a), the *in vitro* ubiquitination assay results indicated that hMEX‐3C Ring finger domain functions as an E3 ligase in combination with specific E2s, such as UbcH5b. We reasoned that the hMEX‐3C Ring finger domain contained a crucial recognition site for the E2. Given that the overall structure of hMEX‐3C Ring finger domain closely resembles the Ring finger domain of MDM2, we speculated that the Ring finger domain of hMEX‐3C may interact with UbcH5b via the same binding site MDM2 used. The interaction between MDM2 Ring finger domain and UbcH5b–Ub showed that several key residues of MDM2/MDMX play a crucial role in stabilizing the closed UbcH5b–Ub conformation.^19^, ^28^ According to the sequence alignment [Fig. 1(a)] and structural superimposition with the complex structure of MDM2–MDMX–UbcH5b–Ub (PDB ID 5MNJ) [Fig. 3(a)], we speculated that several corresponding residues in hMEX‐3C Ring finger domain such as V609, I610, F612, N614, K635, I636, C637, K639, P645, V646, and Q648, may contribute to the interaction with E2–Ub. Moreover, to analyze the roles of predicted interface residues, we mutated them to alanine, respectively. In the self‐ubiquitination assays, mutants of I610A, I636A, P645A, and Q648A abolished activity, while V609A and K639A reduced activity [Fig. 3(b)]. These residues are mainly located at the surface of hMEX‐3C Ring finger domain and may be responsible for the recruitment of E2–Ub. The Residues I440 and R479 in MDM2 play key role to interact with E2^26^ and R479 facilitates to stabilize the closed active E2–Ub conformation.^19^, ^28^ Hence, we identified that I610 and Q648 in hMEX‐3C may play key role to interact with E2 and stabilize ubiquitin in the closed active E2–Ub conformation. [Fig. 3(c)]. Another two residues, V439 and V477 in MDM2, have been deduced to lie at the edge of the E2–Ub binding site.^19^, ^28^ Thus, we also identified V609 and V646 in hMEX‐3C which may locate at the edge of the E2–Ub binding site. To figure out the role of V609 and V646, we mutated these two residues to glutamic acid. When assayed as homodimers both mutants were inactive, indicating the requirement of extended nature of the side chains in residues for functional interaction with E2–Ub [Fig. 3(d)]. Collectively, these results helped us to identify the key residues required for the interaction of hMEX‐3C Ring finger domain with E2–Ub to catalyze ubiquitination.

### *A role for Zn* ^*2+*^ *in the ubiquitination of the Ring finger domain of hMEX‐3C*

**Figure 4:**
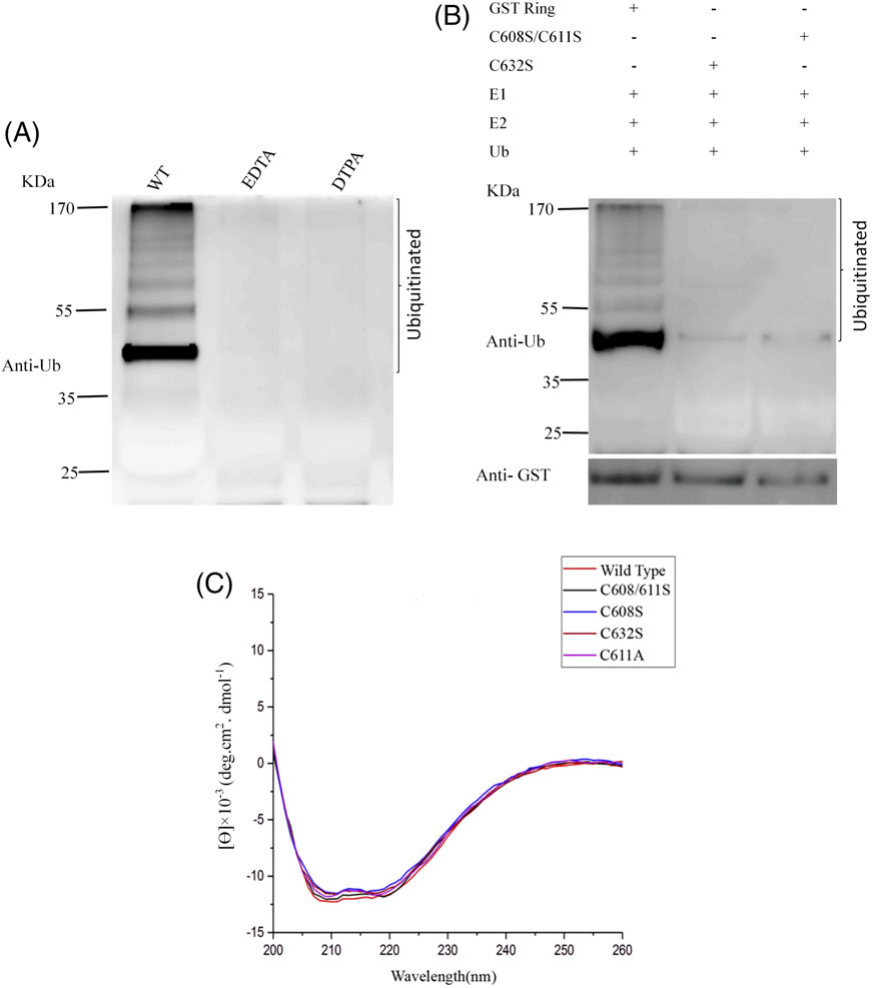
Ubiquitination of the Ring finger domain of hMEX‐3C depends on the intact Ring finger domain and zinc binding. (A) GS bound GST‐hMEX‐3C Ring finger domain was incubated with indicated potent chelators (EDTA and DTPA), respectively, before assaying ubiquitination. (B) Purified Ring finger domain of hMEX‐3C (Lane 1), mutants C632S (Lane 2), and C608S/C611S (Lane 3) was incubated, respectively, in the presence of E2 (UbcH5b), recombinant E1 and ubiquitin. Then the reaction mixtures were fractionated by SDS‐PAGE and analyzed by immunoblotting using anti‐Ub and anti‐GST antibodies to determine ubiquitinated products. (C) CD spectra of hMEX‐3C Ring finger domain and its mutants.

Since zinc coordination is essential to the structure and function of zinc finger domains, we speculated that zinc binding of hMEX‐3C Ring finger domain is needed to mediate ubiquitylation. To evaluate the role for zinc coordination in the ubiquitination of hMEX‐3C Ring finger domain, we used metal‐chelating reagents, such as EDTA and DTPA, to treat the Ring finger domain and observed a loss of activity [Fig. 4(a)]. This result revealed that the eradication of zinc ions by using potent chelators leads to the inactivation of E3 ligase activity mediated by the Ring finger domain of hMEX‐3C. To more carefully evaluate the role of Zn^2+^ binding in the ubiquitination of hMEX‐3C Ring finger domain, we made mutations of zinc‐coordinating cysteine to serine. The two adjacent cysteine residues (C608 and C611) and the distant C632 were mutated to serine, respectively [Fig. 4(b)]. The Mutant C632S and C608/611S almost lost their E3 activity compared with the wild type (WT) [Fig. 4(b), Lane (2,3)]. In addition, CD spectrometry indicated the secondary structures of the mutants were nearly identical to the WT [Fig. 4(c)]. These findings indicated that an intact zinc‐coordinated Ring finger domain of hMEX‐3C is very important to catalyze self‐ubiquitination.

## Discussion

MEX‐3 was first identified as a translational repressor in nematode *Caenorhabditis elegans. C. elegans* MEX‐3 protein is a regulator of translation that specifies the posterior blastomere identity during early embryogenesis and contributes to the maintenance of germline totipotency, immune responses, differentiation, and cancer.^8^, ^12^, ^13^, ^14^, ^15^, ^29^ In mammals, there are four MEX‐3 proteins which are thought to play same roles with that of the *C. elegans* MEX‐3.^15^ MEX‐3C contains two KH domains and one carboxyl‐terminal Ring finger domain. The two KH domains facilitate RNA binding and are important to interact with 3′ UTR of its target *HLA‐A2* mRNA*,* inducing its Ring‐dependent degradation. The Ring finger domain of MEX‐3C has a central role for RNA degradation of *HLA‐A2* mRNA, therefore, modulating natural killer cell activity.^12^, ^17^, ^18^ As it has been reported, hMEX‐3C is responsible for the *in vivo* Lys‐63 linked ubiquitination of RIG‐I and mutation of one cysteine in the Ring finger domain leads to the abolishment of RIG‐I ubiquitination.^10^ Further biochemical experiments indicated that Residues 382–599, the region between the KH domains and the Ring finger domain, binds to RIG‐I.^10^ Combined with the previous study, our results revealed that two parts of hMEX‐3C, Residues 382–599 and 600–652, are involved in the interaction with physiological substrate RIG‐I and implementation of ubiquitination activity, respectively.

Ring finger domains, differing considerably in composition and length, are characterized by seven cysteines and histidine which coordinate two zinc ions. The consensus sequence is CX_2_CX_(9–39)_CX_(1–3)_HX_(2–3)_C/HX_2_CX_(4–48)_CX_2_C.^30^, ^31^ In the first and last three coordination sites, Ring finger domains have cysteines and in the fourth coordination site, Ring finger domains have a histidine. In addition, depending on the fifth coordination site, Ring finger domains can be divided into two canonical RING types, C_3_HC_4_ (RING‐HC) or C_3_H_2_C_3_ RING (RING‐H2). The cysteines/histidine (1st, 2nd, 5th, 6th) coordinate one cation and others (3rd, 4th, 7th, 8th) coordinate the second cation.^31^ Therefore, hMEX‐3C Ring finger domain belongs to the RING‐HC category, as determined by the consensus sequence [Fig. 1(a)].

The Ring finger domain is known for its potential E3 ubiquitin activity, cooperating with E2 to generate polyUb chains.^32^, ^33^ Previous studies have revealed that Ring‐containing protein hMEX‐3C is an E3 ubiquitin ligase *in vivo* that localizes to stress granules (SG) and triggers retinoic acid‐inducible gene‐1 (RIG‐I)‐dependent antiviral responses by RIG‐I ubiquitination.^10^ However, the molecular basis for the ubiquitination catalyzed by hMEX‐3C has not been thoroughly explained. Here, we solved the crystal structure of the Ring finger domain of hMEX‐3C to provide a structural perspective for its ubiquitylating characteristic [Fig. 1(b)]. The Ring finger domain of hMEX‐3C is homodimer in solution and displays a canonical Ring fold, which is very similar to the Ring finger domain of MDM2/MDMX heterodimer [Fig. 1(c)]. It has been reported that dimeric Rings E3 ligase stabilize the closed E2–Ub conjugates by using both Rings in the dimer to interact with ubiquitin of the E2 intermediate^20^, ^23^ and cooperate to facilitate the ubiquitination reaction.^33^, ^34^ In this research, the superimposition of our dimeric Ring structure and the complex of MDM2–MDMX–UbcH5b–Ub indicates there are no steric clashes [Fig. 3(a)] which indicates that the Ring finger domain of hMEX‐3C could bind UbcH5b–Ub to catalyze ubiquitination. To extend our findings, we studied ‘*in vitro’* E3 ligase activity of the Ring finger domain of hMEX‐3C with three different E2s (UbcH5b, UbcH5c, and UBE2H) [Fig. 2(a)] and discovered that the Ring finger domain of hMEX‐3C requires specific E2 such as UbcH5b and UbcH5c to initiate ubiquitination process and behaves as an E3 ubiquitin ligase [Fig. 2(a)].

It has been reported that the residues in the zinc chelating loops and α helix of Ring finger domain are needed for their interaction with E2.^35^ Consistent with these reports, corresponding residues predicted to mediate E2 recruitment in hMEX‐3C Ring finger domain are proven to be crucial to the E3 ligase activity [Fig. 3(b)]. Interestingly, mutations of V609 and V646 to glutamic acid totally abolish the ubiquitination activity of hMEX‐3C Ring finger domain [Fig. 3(d)], which indicates the requirement of extended nature of the surface in regulating the functional interaction with the E2.^19^, ^28^ The ubiquitination assays of hMEX‐3C revealed that the entire Ring finger domain, together with specific E2s, plays a remarkable role in its self‐ubiquitination [Fig. 4(a,b)].

Our results provided first structural insight into the Ring finger domain of hMEX‐3C. Furthermore, our entire experiments showed strong evidence that the Ring finger domain, in the specific molecular context, is a module that cooperates with specific E2s and facilitates ubiquitination. We hope this evidence will help to further clarify the biological function of hMEX‐3C.

## Materials and Methods

### *Cloning, expression, and purification*

The gene encoding the Ring finger domain of hMEX‐3C (Residues 603–659), was amplified by PCR with corresponding primers. The PCR products were purified and digested with NdeI and XhoI. Then, the digested fragment was cloned into pGEX‐TEV vector, a modified vector for the expression of Glutathione S‐transferase (GST) fusion proteins with a proteolytic site. Mutants of Ring finger domain of hMEX‐3C were obtained using Quick Change II Site‐Directed Mutagenesis kit (Agilent).

Plasmids of GST‐hMEX‐3C Ring finger domain and the mutants were transformed into *E. coli.* BL21 (DE3) competent cells. The single colony was inoculated into 5 mL LB medium and incubated at 37°C overnight. The following day, 5 mL strains were then transferred to a 100 mL LB medium for approximately 3 h. The 100 mL strains were finally added to 1 L LB medium with 20 μg/mL ampicillin. Protein expression was induced by 0.4 m*M* isopropyl‐b‐d‐thiogalactopyranoside (IPTG) at 16°C for 20 h. The cells were harvested by centrifugation at 5000 rpm for 10 min and the pelleted cells were resuspended in a lysis buffer (20 m*M* Tris, 1 *M* NaCl at pH 8.0). The cells were lysed by sonication at 4°C followed by centrifugation at 13,000 rpm for 30 min. The protein lysates were purified on a GST column (GE Healthcare) using 1 *M* NaCl, 20 m*M* Tris–HCl (pH 8.0). The eluted proteins were further purified using Highload 16/60 Superdex 75 size exclusion column (GE Healthcare) equilibrated with 500 m*M* NaCl, 20 m*M* Tris–HCl (pH 8.0). The target proteins were analyzed and confirmed by SDS‐PAGE. The SDS‐PAGE analysis showed the presence of a high molecular protein band of about 33 kDa. The mutants were purified by the same procedure as described above.

### *Crystallization, data collection, structure determination, and refinement*

Prior to crystallization, the Ring finger domain of hMEX‐3C was dialyzed against a buffer containing 200 m*M* NaCl, 20 m*M* Tris (pH 8.0). The crystals of hMEX‐3C Ring finger domain were obtained by sitting drop vapor diffusion against 0.2 *M* ammonium acetate, 0.1 *M* Bis–Tris (pH 6.5) and 25% w/v polyethylene glycol 3350 at 18°C with a protein concentration of 15 mg/mL. For the crystals, the mother liquid supplemented with 30% (v/v) glycerol was used as cryoprotectant. Crystals were soaked with the cryoprotectant and then flash‐frozen in liquid nitrogen.

Diffraction data from crystals of hMEX‐3C Ring finger domain were collected on beamline BL19U at Shanghai Synchrotron Radiation Facility (SSRF) at the SSRF and processed with HKL2000. Processing statistics are shown in Table 1. The structure was solved using the single‐wavelength anomalous diffraction (SAD). The positions of 16th zinc atoms were clearly located using SHELXC/D/E, corresponding to two zinc atoms per monomer and eight molecules per asymmetric unit. Electron density maps following density modification in SOLOMON were clearly interpretable and an initial model was built by Arp/Warp. This model was used as a starting point for refinement in REFMAC5, followed by iterative cycles of manual rebuilding in COOT and further refinement. By using PYMOL, structure analysis was performed (http://www.pymol.org).

### *In vitro ubiquitination assay*

For this assay, 1 μg of bacterially produced GST‐hMEX‐3C Ring finger domain WT and mutants were, respectively, incubated at 37°C for 90 min in a reaction mixture with 100 n*M* E1 (Sigma‐Aldrich), 1 μM each of the different E2s (UbcH5b, UbcH5c, and UBE2H, Boston Biochem), 5 μM His_6_ ubiquitin (Sigma‐Aldrich), 5 m*M* ATP, 5 m*M* MgCl_2,_ 5 m*M* DTT, and 50 m*M* Tris–HCl (pH 7.5) and ddH_2_O to a final volume of 10 μL. After dissolved the reactions with 10 μL of 2 × SDS sample loading buffer, products were analyzed by SDS‐PAGE. The reactions were analyzed by western blotting using anti‐Ub and anti‐GST antibodies (Sigma‐Aldrich).

### *Chelation experiment*

To analysis the chelation experiment, GS‐bound proteins were incubated for 18 h at 4°C with three changes of PBS buffer plus 5 m*M* of ethylenediaminetetraacetic acid (EDTA) and disodiumtriaminopentacetic acid (DTPA) potent chelators.

### *Circular dichroism (CD) spectroscopy*

The CD measurements of the Ring finger domain of hMEX‐3C and mutants were implemented on a Jasco‐810 spectropolarimeter. The CD spectra were recorded at wavelengths from 200 to 260 nm using 0.1 cm path length cell and the scan rate is 100 nm min^−1^ at 25°C. The proteins were concentrated to 0.10 mg/mL in 30 m*M* phosphate buffer (pH 8.0). A buffer sample was used as a control.

## Structure Deposition Information

The coordinates for the structure described in this work have been deposited in the Protein Data Bank (PDB) under the Accession code 5ZI6.

## Supporting information


**Fig S1.** Size exclusion chromatography (SEC) of hMEX‐3C Ring finger domain. GST fused MEX‐3C Ring finger domain digested by TEV protease and then loaded into S75 column. The molecular weight of hMEX‐3C Ring finger domain calculated from its elution volume.
