# Supplementary material for: Structural and functional characterization of hMEX‐3C Ring finger domain as an E3 ubiquitin ligase
Source: Protein Sci. 2018 Oct 18;27(9):1661–9. doi: 10.1002/pro.3473 (PMC6194269; doi:10.1002/pro.3473)
Supplement: Supplementary file 1 — Fig S1. Size exclusion chromatography (SEC) of hMEX‐3C Ring finger domain. GST fused MEX‐3C Ring finger domain digested by TEV protease and then loaded into S75 column. The molecular weight of hMEX‐3C Ring finger domain calculated from its elution volume. [file PRO-27-1661-s001.docx]

**Supporting Information**





Fig S1. Size Exclusion Chromatography (SEC) of hMEX-3C Ring finger domain. GST fused MEX-3C Ring finger domain digested by TEV protease and then loaded into S75 column. The molecular weight of hMEX-3C Ring finger domain calculated from its elution volume.
